# Supplementary material for: Effect of Substrate-RF on Sub-200 nm Al0.7Sc0.3N Thin Films
Source: Micromachines (Basel). 2022 May 31;13(6):877. doi: 10.3390/mi13060877 (PMC9230010; doi:10.3390/mi13060877)
Supplement: Supplementary file 1 [file micromachines-13-00877-s001.zip › micromachines-1740749-supplementary.pdf]

micromachines

MDPI

Supplemental Material

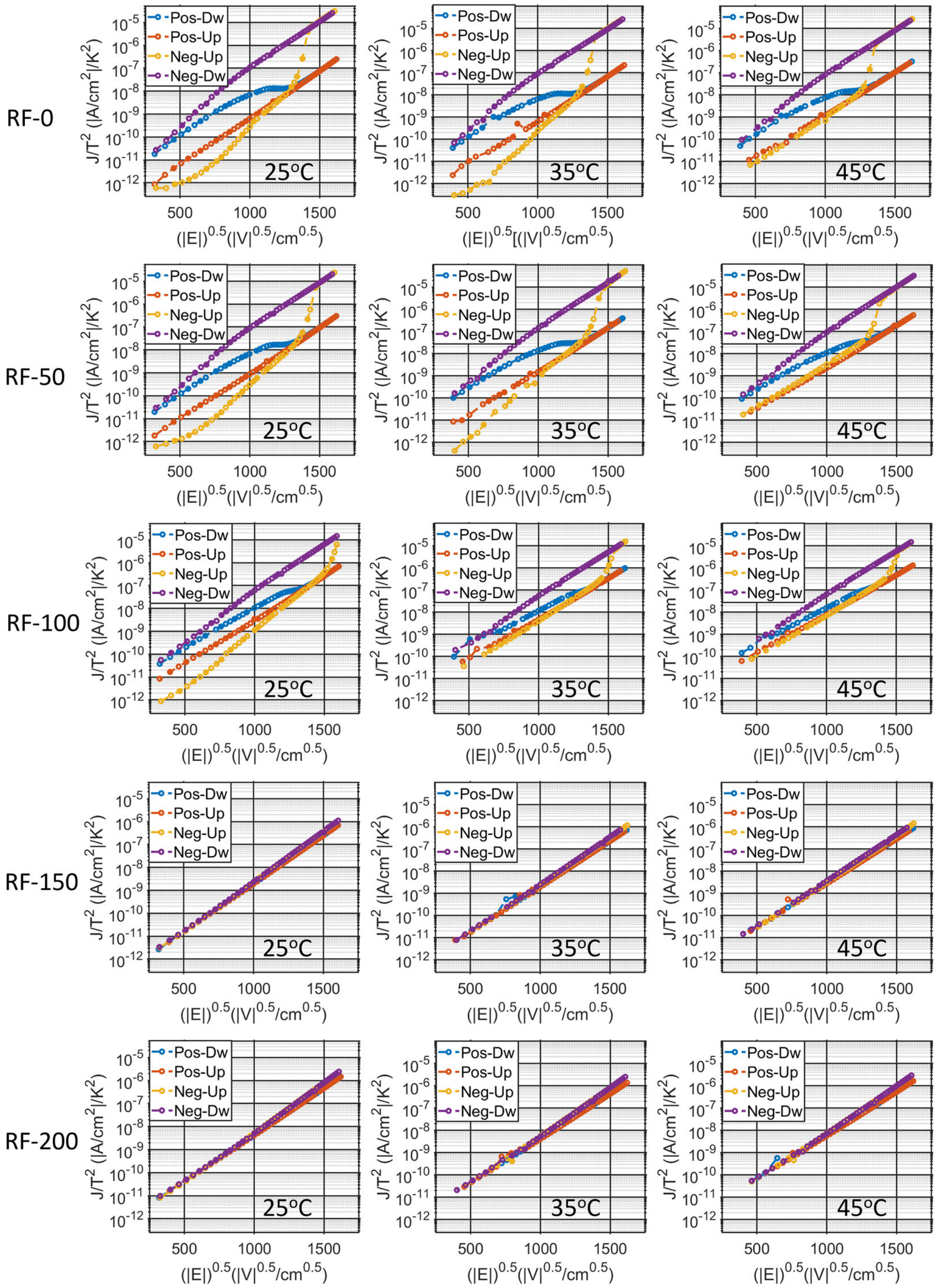

Figure S1. Summary of Schottky-emission plots for all the samples at 3 different temperatures. From top to bottom: RF-0, RF-50, RF-100, RF-150, RF-200, From left to right: 25°C, 35°C, 45°C.

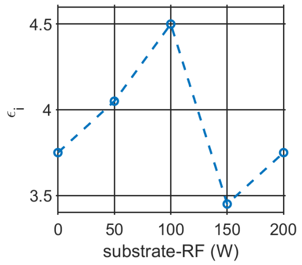

Figure S2. Summary of extracted optical permittivity per different substrate-RF.
The permittivities are extracted from the slopes of the ln(J/T²) vs √E characteristics [16].

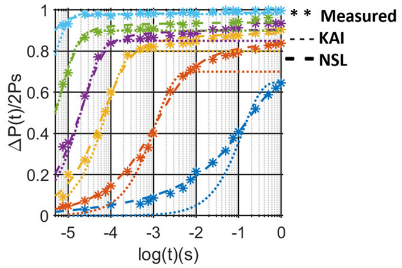

Figure S3. Comparison NSL vs KAI models, confirming a better matching with measured data when the activation time is express though a Lorentzian distribution (NSL) instead of a Delta-Dirac (KAI).

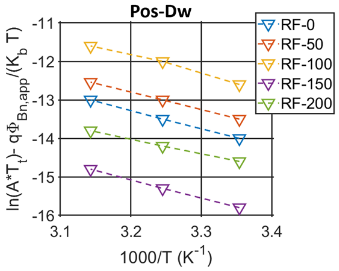

Figure S4. Intercepts variations per different temperatures corresponding to the down-polarity state.
